# Ambient Air Pollution and Autism in Los Angeles County, California

**DOI:** 10.1289/ehp.1205827

**Published:** 2012-12-18

**Authors:** Tracy Ann Becerra, Michelle Wilhelm, Jørn Olsen, Myles Cockburn, Beate Ritz

**Affiliations:** 1 Department of Epidemiology, Fielding School of Public Health, University of California, Los Angeles, Los Angeles, California, USA; 2 Department of Preventive Medicine, Keck School of Medicine, University of Southern California, Los Angeles, California, USA

**Keywords:** air pollution, autism, land-use regression, pregnancy, traffic

## Abstract

Background: The prevalence of autistic disorder (AD), a serious developmental condition, has risen dramatically over the past two decades, but high-quality population-based research addressing etiology is limited.

Objectives: We studied the influence of exposures to traffic-related air pollution during pregnancy on the development of autism using data from air monitoring stations and a land use regression (LUR) model to estimate exposures.

Methods: Children of mothers who gave birth in Los Angeles, California, who were diagnosed with a primary AD diagnosis at 3–5 years of age during 1998–2009 were identified through the California Department of Developmental Services and linked to 1995–2006 California birth certificates. For 7,603 children with autism and 10 controls per case matched by sex, birth year, and minimum gestational age, birth addresses were mapped and linked to the nearest air monitoring station and a LUR model. We used conditional logistic regression, adjusting for maternal and perinatal characteristics including indicators of SES.

Results: Per interquartile range (IQR) increase, we estimated a 12–15% relative increase in odds of autism for ozone [odds ratio (OR) = 1.12, 95% CI: 1.06, 1.19; per 11.54-ppb increase] and particulate matter ≤ 2.5 µm (OR = 1.15; 95% CI: 1.06, 1.24; per 4.68-μg/m^3^ increase) when mutually adjusting for both pollutants. Furthermore, we estimated 3–9% relative increases in odds per IQR increase for LUR-based nitric oxide and nitrogen dioxide exposure estimates. LUR-based associations were strongest for children of mothers with less than a high school education.

Conclusion: Measured and estimated exposures from ambient pollutant monitors and LUR model suggest associations between autism and prenatal air pollution exposure, mostly related to traffic sources.

Autistic disorder (AD) is a serious developmental condition characterized by impairments in social interaction, abnormalities in verbal and nonverbal communication, and restricted stereotyped behaviors thought to be attributable to insults to the developing fetal and/or infant brain (American Psychiatric Association 2000; Geschwind and Levitt 2007). The prevalence of autism has risen for the past 20 years, partly due to changes in case definition and improved case recognition. Hertz-Picciotto and Delwiche (2009) suggested the observed rise in incidence in California between 1990 and 2001 may partially but not fully be explained by younger age at diagnosis (12% increase) and inclusion of milder cases (56% increase). Although evidence for genetic contributions is considered quite strong, twin concordance research recently suggested that environmental causes are also important (Hallmayer et al. 2011), and it is quite conceivable that multiple genes interact with environmental factors (Cederlund and Gillberg 2004; Glasson et al. 2004).

Few studies to date have examined the impact of air pollution on brain development in general during pregnancy, although air pollution exposure during the prenatal period has been associated with a variety of adverse birth outcomes (Ritz and Yu 1999; Ritz et al. 2000; Srám et al. 2005; Williams et al. 1977) and neuropsychological effects later in childhood (Calderón-Garcidueñas et al. 2008; Edwards et al. 2010; Perera et al. 2006, 2012; Suglia et al. 2008; Tang et al. 2008; Wang et al. 2009). The biological mechanisms by which air pollution may cause autism are largely unknown, although the immune system has been implicated as possibly playing a role (Hertz-Picciotto et al. 2008). Only three studies to date have examined associations between autism and air pollution exposures during the prenatal period (Kalkbrenner et al. 2010; Volk et al. 2010; Windham et al. 2006). In one study, autism was associated with ambient air concentrations of chlorinated solvents and heavy metals near birth residences (Windham et al. 2006). Another study of autism reported elevated odds ratios (ORs) for methylene chloride, quinoline, and styrene exposures in ambient air, but near-null effect estimates for ambient air metals and other pollutants (Kalkbrenner et al. 2010). A third study reported that children born to mothers living within 309 m of a freeway during pregnancy were more likely to be diagnosed with autism than children whose mothers lived > 1,419 m from a freeway (Volk et al. 2010).

We derived air pollution exposure measures using data from government air monitoring stations that provide information on spatial and temporal variations in criteria pollutants, and from a land use regression (LUR) model we developed for the Los Angeles Air Basin. The LUR model allowed us to greatly improve our spatial characterization of traffic-related air pollution. Because heterogeneity of the autism phenotype and its severity may be attributable to influences on different critical gestational windows of brain development (Geschwind and Levitt 2007), we also seasonalized these traffic measures to investigate vulnerable trimesters of development. Here we examine associations between measured and modeled exposures to prenatal air pollution and autism in children born to mothers in Los Angeles County, California, since 1995.

## Methods

In this population-based case–control study, our source population consisted of children born in 1995–2006 to mothers who resided in Los Angeles County at the time of giving birth.

*Case ascertainment and definition.* In Los Angeles, children with autism are identified through seven regional centers, contracted by the California Department of Developmental Services (DDS), whose staff determine eligibility and coordinate services in their respective service areas. Cases are children given a primary diagnosis of AD, the most severe among the autism spectrum disorders (ASD) diagnoses, between 36 and 71 months of age at a Los Angeles Regional Center during 1998–2009. During our study period, eligibility for DDS services did not depend on citizenship or financial status—services were available to all children regardless of socioeconomic, health insurance status, or racial/ethnic identification. Referrals to the regional centers are usually made by pediatricians, other clinical providers, and schools, but parents may also self-refer their children.

The diagnosis of AD was based on the *Diagnostic and Statistical Manual of Mental Disorders, 4th Edition, Text Revision* (DSM-IV-R) (American Psychiatric Association 2000), code 299.00, reported on the Client Development Evaluation Report (CDER). Validation studies have established the reliability and validity of the CDER in California (California Department of Developmental Services 1986, 2007).

*Record linkage.* We attempted to link 10,821 DDS records of children with autism to their respective birth records using the National Program of Cancer Registries Registry Plus^TM^ Link Plus Software [Centers for Disease Control and Prevention (CDC) 2010a]. Given the child’s first and last name, birth date, and sex; mother’s first and last name and birth date; and father’s last name and birth date, we probabilistically matched the two records and reviewed all high scoring linkages (≥ 25), almost half of the linkages (9,120 of 22,806), only accepting those manually confirmed to be likely matches (see CDC for record linkage concepts) (CDC 2010b). The remaining lower scoring linkages were reviewed using SAS version 9.2 (SAS Institute Inc., Cary, NC) and accepted on the condition that the child’s first and last name, and birth date matched perfectly. We correctly linked 8,600 DDS records (79.5% of all cases) to birth records. Of the 2,221 DDS records not linked to CA birth records, 35% were not born in Los Angeles County, 46% were missing birthplace information, and only 19% recorded the child as born in Los Angeles County. The most common reason for nonlinkage was missing or incomplete linkage information on either of the records.

From among linked cases, we further excluded children whose mother’s residency was outside of Los Angeles County during her pregnancy (*n* = 41), records with missing or implausible gestational ages (< 21 or > 46 weeks) or birth weights (< 500 g or > 6,800 g) (*n* = 508), and cases who did not have a primary diagnosis of AD (*n* = 448), leaving a final sample of 7,603 children with autism successfully linked to a birth certificate who met all inclusion criteria.

*Control selection.* We selected 10 controls for each case from our source population. Using birth certificates, each control was randomly selected without replacement and matched on birth year and sex. In addition, each control’s gestational age at birth had to be equal to or greater than the gestational age at birth of their matched case to ensure prenatal exposures could be estimated for comparable lengths of time. Children were eligible as controls if they had no documentation of autism—did not have a DDS record in Los Angeles County by 2009, had a plausible gestational age (21–46 weeks inclusive) and birth weight (500–6,800 g inclusive), and the mother resided in Los Angeles County at the time of birth.

Matching by birth year balanced the large increase in autism rates during the case ascertainment period, 1998–2009. The matched control set included 76,030 children born during 1995–2006. From among these, we further excluded 248 control children who died before 6 years of age (71 months) based on California death records, leaving 75,782 controls.

Residential locations at delivery that were reported on birth certificates were mapped using a custom geocoder (Goldberg et al. 2008), and further exclusions were necessary if residential addresses were not geocodable (9 cases, 147 controls) [see Supplemental Material, Table S1 (http://dx.doi.org/10.1289/ehp.1205827)]. The geocoded residential locations at birth were then linked to the nearest government air monitoring station in Los Angeles County and our LUR model.

This research was approved by the University of California, Los Angeles, Office of the Human Research Protection Program and the California Committee for the Protection of Human Subjects, and was exempted from informed consent requirements.

*Exposure assessment.* Using measurements for the criteria pollutants carbon monoxide (CO), nitrogen dioxide (NO_2_), nitric oxide (NO), ozone (O_3_), and particulate matter concentrations with an aerodynamic diameter ≤ 10 µm (PM_10_) and ≤ 2.5 µm (PM_2.5_) from nearest monitoring stations, we estimated average exposures for the entire pregnancy and for three specific periods during pregnancy based on the birth date and gestational age reported on the birth certificate: first trimester (estimated first day of last menstrual period through day 92), second trimester (days 93–185), and third trimester (day 186 to date of birth). The length of each pregnancy averaging period for controls was the same as for their matched case: Averaging periods for each autistic risk set were truncated at the gestational age of the matched case at birth. Hourly measurements for CO, NO_2_, NO, and O_3_ (1000–1800 hours) were first averaged for each day if sufficient data were available [for details, see Supplemental Material, Table S2 (http://dx.doi.org/10.1289/ehp.1205827)]. Daily averages for the gaseous pollutants and 24-hr measurements of PM_10_ and PM_2.5_ (collected every 6 and 3 days, respectively) were then averaged over the different pregnancy periods when data were sufficient to do so (see Supplemental Material, Table S2).

To classify prenatal exposures to traffic-related pollutants on a more spatially-resolved scale, we extracted NO and NO_2_ concentration estimates at each residential location from the LUR model surfaces we developed for the Los Angeles Air Basin (Su et al. 2009). This LUR model was based on approximately 200 measurements of outdoor air pollution taken during 2006–2007 in locations across Los Angeles County, in addition to predictors of traffic exhaust concentrations (such as traffic counts, truck routes, and roadways). The model explained 81% and 86% of the variance in measured NO and NO_2_ concentrations, respectively (Su et al. 2009).

The LUR models most closely approximate annual average concentrations. Thus, in addition to using the LUR annual average (“unseasonalized”) estimates, we also generated “seasonalized” estimates to incorporate yearly and monthly air pollution variations. Specifically, using ambient air monitoring data for NO and NO_2_ at the closest monitoring station, the LUR estimates were adjusted to represent pregnancy month–specific LUR values by multiplying the LUR (unseasonalized) estimates for NO and NO_2_ by the ratio of average ambient NO and NO_2_ during each pregnancy month to annual average ambient NO and NO_2_ (2006–2007). These seasonalized monthly LUR values were then averaged over each pregnancy period. We applied the same exclusion criteria for missing values as described above when generating the pregnancy month scaling factors using the government monitoring data.

*Statistical analysis.* We calculated Pearson’s correlation coefficients to examine relations between the various pollutant measures. Associations between air pollution exposure and odds of AD diagnosis were examined using one- and two-pollutant models. We adjusted for LUR estimates of traffic-related exposures in our monitor-based pollutant models and assessed particles and the gaseous pollutant ozone together in the same model. We calculated ORs and 95% CIs using conditional logistic regression to estimate increases in odds of AD per interquartile range (IQR) increase in pregnancy exposures, based on exposure distributions in the controls.

We adjusted for potential confounders for which data were available on birth certificates based on prior knowledge (see Table 1 for categories used in models): maternal age, maternal place of birth, race/ethnicity, and education; type of birth (single, multiple), parity; insurance type (public, private, or other, a proxy for socioeconomic status); and gestational age at birth (weeks). In addition, we estimated pollutant effects without adjustment for gestational age to allow for the possibility that this factor might be an intermediate and thus on the causal pathway between air pollution and autism.

**Table 1:** Demographic and prenatal characteristics by case (7,594) and control group (*n* = 75,635) [*n* (%)].

Characteristics	AD cases	Controlsa
Sex
Male	6,291 (82.8)	62,643 (82.8)
Female	1,303 (17.2)	12,992 (17.2)
Birth year
1995	277 (3.7)	2,762 (3.7)
1996	319 (4.2)	3,173 (4.2)
1997	382 (5.0)	3,812 (5.0)
1998	487 (6.4)	4,859 (6.4)
1999	455 (6.0)	4,533 (6.0)
2000	594 (7.8)	5,904 (7.8)
2001	732 (9.6)	7,285 (9.6)
2002	885 (11.7)	8,776 (11.6)
2003	1,035 (13.6)	10,336 (13.7)
2004	1,034 (13.6)	10,284 (13.6)
2005	874 (11.5)	8,735 (11.6)
2006	520 (6.9)	5,176 (6.8)
Gestational age (weeks) (mean ± SD)	39.0 ± 2.6	39.4 ± 2.3
Maternal characteristics
Maternal age at delivery (years)		
≤ 18	178 (2.3)	4,997 (6.6)
19–25	1,673 (22.0)	23,906 (31.6)
26–30	2,034 (26.8)	20,228 (26.7)
31–35	2,159 (28.4)	16,845 (22.3)
> 35	1,550 (20.4)	9,654 (12.8)
Missing	0	5 (0.0)
Maternal birthplace		
U.S.-born	3,544 (46.7)	32,590 (43.1)
Foreign-born	4,038 (53.2)	42,930 (56.8)
Unknown	12 (0.1)	115 (0.1)
Maternal race/ethnicity		
Non-Hispanic white	2,625 (34.6)	20,616 (27.3)
Non-Hispanic black	622 (8.2)	6,028 (8.0)
Hispanic	3,183 (41.9)	40,118 (53.0)
Asian	1,073 (14.1)	8,123 (10.7)
Other/unknown	91 (1.2)	750 (1.0)
Maternal education		
< High school	1,725 (22.7)	27,232 (36.0)
High school	1,861 (24.5)	20,115 (26.6)
> High school	3,926 (51.7)	27,400 (36.2)
Unknown	82 (1.1)	888 (1.2)
Prenatal characteristics
Type of birth		
Single	7,218 (95.0)	73,880 (97.7)
Multiple	376 (5.0)	1,755 (2.3)
Insurance type		
Public (Medi-Cal)	2,971 (39.1)	39,382 (52.1)
Private	4,432 (58.4)	33,746 (44.6)
Other	117 (1.5)	1,925 (2.6)
Unknown	74 (1.0)	582 (0.8)
Parity		
One (index birth)	3,280 (43.2)	29,399 (38.9)
Two	2,556 (33.7)	23,495 (31.1)
Three	1,134 (14.9)	13,296 (17.6)
> Three	623 (8.2)	9,417 (12.4)
Unknown	1 (0.0)	28 (0.0)
Birth weight (g) (mean ± SD)	3321.0 ± 640.9	3377.8 ± 543.3
Paternal age at delivery (years)
≤ 18	53 (0.7)	1,484 (2.0)
19–25	1,017 (13.4)	16,067 (21.2)
26–30	1,545 (20.4)	17,752 (23.5)
31–35	1,999 (26.3)	17,174 (22.7)
> 35	2,502 (32.9)	17,286 (22.9)
Unknown	478 (6.3)	5,872 (7.8)
Paternal education
< High school	1,508 (19.9)	23,653 (31.3)
High school	1,931 (25.4)	19,725 (26.1)
> High school	3,589 (47.3)	25,145 (33.2)
Unknown	566 (7.4)	7,112 (9.4)
aControls are matched to cases by sex and birth year, and at minimum reached the gestational age of the case.		

We expected maternal education to correlate with estimates of air pollution and autism (Ponce et al. 2005), so we also used unconditional logistic regression models to estimate associations stratified by maternal education (less than high school, high school, more than high school) controlling for the matching variables (birth year, sex, and gestational weeks at birth) in addition to the other covariates noted above.

## Results

Both mothers and fathers of children with autism were older and more educated than parents of control children, and mothers were more often non-Hispanic white but less often Hispanic, especially foreign-born Hispanic (Table 1). A higher percentage of mothers of case children were primiparous and had multiple gestations. As expected, children with autism had a lower mean gestational age at birth and birth weight than control children. Of the children with autism not linked to a Los Angeles County birth record, parental characteristics were undetermined because of frequent missing information—50–60% missing maternal and paternal age/birthday (results not shown). However, of these nonlinked DDS records, 42% of families were Hispanic (results not shown), comparable to the 41.9% of Hispanic mothers of case children included in this study (Table 1).

Unseasonalized LUR-based exposure estimates for NO and NO_2_ were negatively correlated with entire pregnancy ozone (*r* = –0.23 and –0.33, respectively) but positively correlated with entire pregnancy CO, NO, NO_2_, and PM_2.5_ (*r* = 0.22–0.43), and as expected, correlations between measured levels of pollutants and seasonalized LUR estimates were stronger than correlations with unseasonalized LUR estimates (*r* = 0.30–0.73) [see Supplemental Material, Table S3 (http://dx.doi.org/10.1289/ehp.1205827)]. Even though all trimester-specific measures correlated moderately with entire pregnancy averages (*r* ≥ 0.46), second-trimester exposure averages correlated most strongly with entire pregnancy averages (*r* ≥ 0.80), and first- and third-trimester averages for the same pollutants were least correlated (*r* = 0.05–0.37) (results not shown).

We estimated 4–7% relative increases in odds of an AD diagnosis per IQR increase in unseasonalized LUR measures of NO and NO_2_ in adjusted models (Table 2). These OR estimates remained similar (1.03 to 1.09) in two-pollutant adjusted models (Table 3). ORs for autism per IQR increase in monitor-based estimates of entire pregnancy exposure to NO and NO_2_ were slightly smaller than associations with IQR increases in LUR-based estimates (Table 2). We also estimated increases in odds of AD diagnosis per IQR increase in entire pregnancy exposure to ozone (OR = 1.06; 95% CI: 1.01, 1.12) and PM_2.5_ (OR = 1.07; 95% CI: 1.00, 1.15) (Table 2). In two-pollutant models these estimates increased (O_3_ OR = 1.12; 95% CI: 1.06, 1.19; PM_2.5_ OR = 1.15; 95% CI: 1.06, 1.24) when we mutually adjusted for both pollutants (Table 3). In addition, without adjustment for gestational weeks at birth, associations increased further or remained the same; for the two-pollutant models including ozone and PM_2.5_ (O_3_ OR = 1.14; 95% CI: 1.10, 1.19; PM_2.5_ OR = 1.15; 95% CI: 1.09, 1.22) or O_3_ and LUR–NO_2_ (O_3_ OR = 1.10; 95% CI: 1.06, 1.14; LUR–NO_2_ OR = 1.10; 95% CI: 1.07, 1.13) (results not shown).

**Table 2:** Associations between IQR increases in entire pregnancy average air pollution exposures and AD: conditional logistic regression analysis using matched controls.*^a^*

Exposure metric	IQR	Unadjusted	Adjustedb	
		OR	nc (case/control)	OR (95%CI)
U-LUR-NO	9.40 ppb	0.87	7,420/72,231	1.04 (1.00, 1.08)
U-LUR-NO2	5.41 ppb	0.91	7,420/72,231	1.07 (1.03, 1.12)
S-LUR-NO	18.46 ppb	0.84	6,279/52,144	1.02 (0.96, 1.08)
S-LUR-NO2	9.70 ppb	0.87	6,279/52,144	1.05 (0.98, 1.12)
CO	0.55 ppm	0.85	7,421/72,253	0.99 (0.94, 1.05)
NO	29.67 ppb	0.85	7,421/72,253	1.01 (0.95, 1.07)
NO2	10.47 ppb	0.89	7,421/72,253	1.04 (0.98, 1.10)
O3	11.54 ppb	1.19	7,421/72,253	1.06 (1.01, 1.12)
PM10	8.25 µg/m3	0.96	6,795/63,662	1.03 (0.96, 1.10)
PM2.5	4.68 µg/m3	1.01	5,840/55,776	1.07 (1.00, 1.15)
Abbreviations: S-LUR, seasonalized land use regression; U-LUR, unseasonalized land use regression. aControls matched to cases by birth year, sex, and at minimum reached the gestational age of the case. bAdjusted for maternal age, education, race/ethnicity, maternal place of birth; type of birth, parity, insurance type, gestational weeks at birth (continuous). cSample with complete data (i.e., strata with at least one case and one control).				

**Table 3:** Associations between IQR increases in entire pregnancy average air pollution exposures and AD: conditional logistic regression analysis using matched controls,a adjustedb two-pollutant models.

Pollutant 1	IQR	Pollutant 2	IQR	nc (case/control)	Pollutant 1 OR (95%CI)	Pollutant 2 OR (95%CI)
O3	11.54 ppb	U-LUR-NO	9.4 ppb	7,420/72,231	1.08 (1.03, 1.14)	1.06 (1.02, 1.11)
O3	11.54 ppb	U-LUR-NO2	5.4 ppb	7,420/72,231	1.08 (1.03, 1.14)	1.09 (1.04, 1.13)
NO	29.67 ppb	U-LUR-NO	9.4 ppb	7,420/72,231	0.99 (0.93, 1.05)	1.04 (1.00, 1.09)
NO	29.67 ppb	U-LUR-NO2	5.4 ppb	7,420/72,231	0.98 (0.92, 1.04)	1.08 (1.03, 1.13)
CO	0.55 ppm	U-LUR-NO	9.4 ppb	7,420/72,231	0.97 (0.92, 1.03)	1.05 (1.00, 1.09)
CO	0.55 ppm	U-LUR-NO2	5.4 ppb	7,420/72,231	0.96 (0.91, 1.02)	1.08 (1.03, 1.13)
PM10	8.25 µg/m3	U-LUR-NO	9.4 ppb	6,794/63,642	1.02 (0.95, 1.10)	1.04 (1.00, 1.09)
PM10	8.25 µg/m3	U-LUR-NO2	5.4 ppb	6,794/63,642	1.00 (0.93, 1.07)	1.08 (1.03, 1.13)
PM2.5	4.68 µg/m3	U-LUR-NO	9.4 ppb	5,839/55,757	1.06 (0.99, 1.14)	1.03 (0.98, 1.08)
PM2.5	4.68 µg/m3	U-LUR-NO2	5.4 ppb	5,839/55,757	1.05 (0.97, 1.12)	1.07 (1.01, 1.12)
O3	11.54 ppb	PM10	8.25 µg/m3	6,795/63,662	1.06 (1.01, 1.12)	1.04 (0.97, 1.11)
O3	11.54 ppb	PM2.5	4.68 µg/m3	5,840/55,776	1.12 (1.06, 1.19)	1.15 (1.06, 1.24)
U-LUR, unseasonalized land use regression. aControls matched to cases by birth year, sex, and at minimum reached the gestational age of the case. bAdjusted for maternal age, education, race/ethnicity, maternal place of birth; type of birth, parity, insurance type, gestational weeks at birth (continuous). cSample with complete data (i.e., strata with at least one case and one control).						

In general, effect estimates did not show consistent patterns across trimesters in one-pollutant models. For example, average second- and third- but not first-trimester exposures to O_3_ were associated with AD [first-trimester OR = 1.00 (95% CI: 0.97, 1.03); second-trimester OR = 1.02 (95% CI: 1.00, 1.05); third-trimester OR = 1.04 (95% CI: 1.01, 1.06)] [see Supplemental Material, Table S4 (http://dx.doi.org/10.1289/ehp.1205827)].

Adjusting for maternal education changed air pollution effect estimates most strongly, likely because socioeconomic status is strongly associated both with air pollution exposure and autism diagnosis. We also investigated potential effect measure modification of the air pollution and autism association: We examined whether air pollution effect estimates vary according to strata of maternal education possibly due to differences in vulnerability, in actual exposure, or exposure and outcome misclassification. Generally, LUR-based traffic-related pollutant estimates showed the strongest association with autism in children of the least educated mothers, compared with mothers in the highest educational stratum (Table 4).

**Table 4:** Associations between IQR increases in entire pregnancy average air pollution exposures and AD: unconditional logistic regression by maternal education.

Pollutant	IQR	Adjusted ORs by maternal educationa					
		< High school		High school		> High school	
		Case/control	Adjusted OR	Case/control	Adjusted OR	Case/control	Adjusted OR
U-LUR-NO	9.40 ppb	1,713/27,051	1.11 (1.05, 1.18)	1,842/19,962	1.03 (0.97, 1.09)	3,865/26,987	0.99 (0.95, 1.03)
U-LUR-NO2	5.41 ppb	1,713/27,051	1.17 (1.10, 1.25)	1,842/19,962	1.06 (1.00, 1.13)	3,865/26,987	1.03 (0.99, 1.07)
S-LUR-NO	18.46 ppb	1,435/23,270	1.03 (0.96, 1.10)	1,513/16,533	1.02 (0.95, 1.09)	3,331/22,872	1.01 (0.96, 1.07)
S-LUR-NO2	9.70 ppb	1,435/23,270	1.04 (0.97, 1.27)	1,513/16,533	1.07 (0.99, 1.15)	3,331/22,872	1.07 (1.01, 1.12)
CO	0.55 ppm	1,714/27,036	0.90 (0.85, 0.96)	1,842/19,949	1.03 (0.97, 1.09)	3,865/26,960	1.09 (1.04, 1.14)
NO	29.67 ppb	1,714/27,036	0.96 (0.89, 1.03)	1,842/19,949	1.02 (0.95, 1.09)	3,865/26,960	1.04 (0.99, 1.10)
NO2	10.47 ppb	1,714/27,036	0.97 (0.90, 1.04)	1,842/19,949	1.08 (1.01, 1.16)	3,865/26,960	1.07 (1.02, 1.12)
O3	11.54 ppb	1,714/27,036	1.09 (1.02, 1.16)	1,842/19,949	1.07 (1.01, 1.14)	3,865/26,960	1.04 (0.99, 1.09)
PM2.5	8.25 µg/m3	1,352/20,540	1.04 (0.96, 1.12)	1,415/15,547	1.09 (1.01, 1.17)	3,074/21,970	1.06 (1.00, 1.12)
PM10	4.68 µg/m3	1,585/24,775	0.97 (0.91, 1.04)	1,670/18,273	1.08 (1.01, 1.16)	3,550/24,707	1.02 (0.97, 1.07)
Abbreviations: S-LUR, seasonalized land use regression; U-LUR, unseasonalized land use regression. Missing maternal education (case/control): U-LUR: 63/718; S-LUR: 50/605; monitor-based criteria: 63/715; PM10: 57/659; PM2.5: 51/596. aAdjusted for child’s birth year, sex; maternal age, race/ethnicity, maternal place of birth; type of birth, parity, insurance type, gestational weeks at birth (continuous).							

## Discussion

We estimated an approximately 3–9% relative increase in the odds of AD per IQR increase in entire pregnancy exposure to NO (9.40 ppb) and NO_2_ (5.41 ppb) as estimated by our two-pollutant LUR models. Our LUR model was built upon neighborhood-level measures of nitrogen oxides (NO_x_) and represents smaller-scale variability in exhaust pollutants, compared with estimates based on air monitoring station measurements (Zhou and Levy 2007). We also estimated a 5–15% relative increase in the odds of AD per IQR increase in entire pregnancy exposure to PM_2.5_ (4.68 μg/m^3^) (Table 3), a pollutant whose concentrations are driven partly by fossil fuel combustion in motor vehicles. In addition, an 11.54-ppb increase in O_3_ exposures during pregnancy was associated with a 6–12% relative increase in the odds of having a child diagnosed with autism.

Few studies have previously examined associations between air pollution–related exposures during the prenatal period and later development of autism, and none used ambient air monitoring data or LUR models to estimate risk in a large population. A relatively small study (284 cases, 657 controls) in the San Francisco Bay, California, area used study-specific census tract pollution scores derived from annual average concentrations and found hazardous air pollutant (HAP) concentrations (i.e., mercury, cadmium, nickel, trichloroethylene, and vinyl chloride) near birth residences to be associated with autism (Windham et al. 2006). A study by Kalkbrenner et al. (2010) in North Carolina and West Virginia, with less exposure variability compared with California, reported near-null effect estimates for metals and several pollutants associated with AD in the San Francisco study. Both studies relied on the same HAP pollutant data source and the CDC autism surveillance system (Autism and Developmental Disabilities Monitoring Network) to identify cases. However, instead of sampling controls from birth certificates, the North Carolina/West Virginia study investigators, using education records, selected control children with speech and language impairment (383 cases, 2,829 controls). A third study (304 autism cases and 259 typically developing controls) based in California [Childhood Autism Risks from Genetics and the Environment (CHARGE) study] reported relatively strong associations (OR = 1.86, 95% CI: 1.04, 3.45) between childhood autism and proximity (living within 309 m) to a freeway during pregnancy (Hertz-Picciotto et al. 2006; Volk et al. 2010). Trimester-specific addresses were geocoded, and measures of distance to freeways and major roads were calculated using geographic information system software. This small study was the first to suggest that traffic-related exposures might increase the risk of autism. In our study, we observed weaker associations with monitor-based and modeled air pollution exposure estimates in a much larger study population.

Gestational toxicity may plausibly result from maternal exposure to NO_2_, which has been shown to disturb early neuromotor development in animals, causing coordination deficits and reduced activity and reactivity in rats (Tabacova et al. 1985); specifically, NO_2_ exposure at low (0.05–0.10 mg/m^3^) and high (1 and 10 mg/m^3^) concentrations for 6 hr each day throughout gestation affected neuromotor development in offspring. The mean NO_2_ level in our study (30.8 ppb) [see Supplemental Material, Table S3 (http://dx.doi.org/10.1289/ehp.1205827)] falls within the exposure range classified as “low” in this animal study (0.05–0.10 mg/m^3^ or 26.6–53.2 ppb). Beckerman et al. (2008) suggested that NO may be a proxy measure for ultrafine particle (UFP; < 0.1 μm in aerodynamic diameter) exposures from traffic exhaust and reported strong correlations between 1-week average concentrations of NO, NO_2_, and NO_x_ and short-term (10 min) measures of UFP (*r* = 0.8–0.9) at varying distances from a major expressway in Toronto, Canada. Fine particles (PM_2.5_) can cause oxidative stress, and *in vitro* animal and human postmortem brain studies showed they can trigger cellular toxicity and brain cell pathology (Lai et al. 2005; Li et al. 2003, Peters et al. 2006). Hertz-Picciotto et al. (2005) found that maternal PM_2.5_ exposures 2 weeks before birth were associated with altered lymphocyte immunophenotypes, and suggested that this might mediate effects of air pollution on childhood morbidity. Developmental immune system disruption has been hypothesized to play a role in neurobehavioral disorders such as autism, considering the close connection between the development of the immune system and the central nervous system (Hertz-Picciotto et al. 2008).

To our knowledge, this is the first study to suggest associations between ozone and AD. Although O_3_ levels have dropped over the last decade, the Los Angeles region still often has the highest levels of O_3_ nationwide, violating federal health standards an average of 137 days/year (averages from 2007 through 2009) (Roosevelt 2011). In contrast with the traffic-related and particle associations that became positive only when we adjusted for maternal education, O_3_ effect estimates moved closer toward the null after adjustment for covariates. This is consistent with expectations, because traffic-related pollution is higher in lower-SES (socioeconomic status) neighborhoods, whereas O_3_ levels are higher in suburban high-SES areas, and autism is more likely to be diagnosed earlier in children of mothers with higher SES. Specifically, O_3_ and NO follow opposite distribution patterns across the Los Angeles Air Basin. O_3_ is formed by photochemical reactions in the presence of precursor pollutants from exhaust, and concentrations are low near freeways/roadways (due to presence of strong NO emission sources) and higher in suburban neighborhoods (Wilhelm et al. 2009). Controlled animal studies suggest that O_3_ may cause adverse neurobehavioral effects after gestational exposure (Kavlock et al. 1980; Petruzzi et al. 1995; Sorace et al. 2001).

We relied on information recorded on California birth certificates to adjust for potential confounding by prenatal risk factors for autism reported in the literature (Gardener et. al. 2009, 2011)—parental age at birth, parity, maternal place of birth, and multiple births. However, we were unable to control for potential confounding due to maternal physical and mental health history, or maternal active or passive smoking. Women giving birth in Los Angeles are predominantly Hispanic, and our survey of 2,543 women giving birth in Los Angeles County in 2003 found that only 1% of foreign-born Hispanic, 5% of U.S.-born Hispanics, and 7% of non-Hispanic whites were active smokers during pregnancy (Hoggatt et al. 2012). Also, a recent study found no association [prevalence ratio = 0.88 (95% CI: 0.72, 1.08)] of maternal smoking during pregnancy with AD (Kalkbrenner et al. 2012). Confounding by other SES-related factors potentially correlated with air pollution is also a concern. Families of lower SES are more likely exposed to air pollution, and less likely represented in the autism case group, possibly due to underascertainment (Durkin et al. 2010; Grineski et al. 2007; Institute of Medicine 1999), which could have potentially biased our effect estimates toward the null. However, we estimated stronger associations among those with the lowest maternal education for LUR-based estimates of NO and NO_2_. We adjusted for type of insurance (public vs. private pay), as well as other SES indicators important in the Los Angeles community (i.e., maternal place of birth and education) because we previously showed that these factors were sufficient to adjust adequately for SES in Los Angeles County birth outcome and air pollution studies; effect estimates for air pollution and birth outcomes were very similar when we adjusted for maternal occupation, income, and education or simply for birth certificate–derived SES measures (Hoggatt et al. 2012).

In addition to being a confounder, gestational age at birth may also be a mediator between air pollution and autism. In analyses not adjusting for gestational weeks at birth we estimated larger or similar effect sizes. However, not adjusting for gestational age at birth may also result in biased estimates because of our matching design. Specifically, because controls were sampled from among children who at birth had reached at minimum the gestational age of the matched case, gestational age as a matching variable required that we analytically control for it. Thus the magnitude and direction of any potential bias from adjusting or not adjusting for gestational age at birth is not easily quantifiable.

A source of exposure measurement error is the reliance on address information reported on birth certificates, which does not account for women who worked far from home or residential mobility during pregnancy. Previous U.S.-based studies (1997–2004) indicate that 15–30% of women change residence during pregnancy (Chen et al. 2010; Lupo et al. 2010). In our previous population-based survey of 2,543 women residing in 111 ZIP codes in Los Angeles County and delivering in 2003, 22% reported moving during pregnancy (Ritz et al. 2007). Our survey also found pregnant women of lower SES less likely to be employed and more likely to spend time near their residence, suggesting exposure is less misclassified for lower compared with higher-SES women.

Distance from a monitoring station likely introduced some nondifferential misclassification of exposure, especially for pollutants such as CO and NO_2_ that are more heterogeneously distributed. On average, the distance between home addresses and the nearest monitoring station was 6.7 miles in our study, and monitor-based estimates of CO, NO, and NO_2_ are questionable in their validity if air pollution measurements are more accurate representations of actual exposures for women living closer to a station (Ghosh et al. 2012; Wilhelm et al. 2011). Ambient station measures for PM_2.5_ and O_3_, however, are less likely to misrepresent actual exposures, because these pollutants are generally considered more homogeneously distributed over larger regions.

LUR-derived NO and NO_2_ estimates are much more spatially resolved than monitor-based estimates, and were previously associated with adverse pregnancy outcomes in the same Los Angeles population (Ghosh et al. 2012; Wilhelm et al. 2011). Our LUR model not only represents local traffic-related pollution well, it reduces possible confounding by spatial SES factors. For example, autism diagnoses have been reported to vary spatially in California due to SES (Van Meter et al. 2010), but measures of air pollution are not inherently influenced by these spatial factors related to SES (Wilhelm et al. 2009). For pollutants that are more homogeneous over larger regional areas, such as PM_2.5_ and O_3_, confounding due to SES is possible; nevertheless, associations were stronger when we mutually adjusted for both pollutants.

A major strength of our study was the use of our novel LUR exposure measures for traffic-related pollution in addition to routine, government monitoring station data for criteria pollutants to help identify specific emissions of concern for autism. Furthermore, selection bias due to participation is unlikely to have occurred.

## Conclusions

The observed association with the LUR model estimates and monitoring station–based O_3_ and PM_2.5_ measures suggest a link between AD and traffic-related exposures during pregnancy. Ideally, future autism and air pollution studies should use neighborhood-level monitoring or modeling of air toxins such as polycyclic aromatic hydrocarbons and possibly speciated PM_2.5_ to determine whether these results are reproducible with improved air pollution assessment.

## Supplemental Material

(541 KB) PDF
