# Supplementary material for: Ambient Air Pollution and Autism in Los Angeles County, California
Source: Environ Health Perspect. 2012 Dec 18;121(3):380–6. doi: 10.1289/ehp.1205827 (PMC3621187; doi:10.1289/ehp.1205827)
Supplement: (541 KB) PDF [file ehp.1205827.s001.pdf]

Supplemental Material:

Ambient Air Pollution and Autism in Los Angeles County, California

Tracy Ann Becerra[1], Michelle Wilhelm[1], Jørn Olsen[1], Myles Cockburn[2], Beate Ritz[1]

[1] Department of Epidemiology, Fielding School of Public Health, University of California, Los Angeles, California, USA

[2] Department of Preventive Medicine, Keck School of Medicine, University of Southern California, California, USA

Table of Contents

Supplemental Material, Table S1:	Results of birth certificate residential address geocoding	Pg. 2
Supplemental Material, Table S2:	Exclusion criteria used when estimating pregnancy period specific monitor-based air pollution exposure metrics	Pg. 3
Supplemental Material, Table S3:	Pollutant distributions and Pearson's correlation coefficients for entire pregnancy averages in controls	Pg. 4
Supplemental Material, Table S4:	Associations between trimester average air pollution exposure during pregnancy and Autistic Disorder, conditional logistic regression analysis using matched controls	Pg. 5

Supplemental Material, Table S1. Results of birth certificate residential address geocoding.

Mapping level	Autistic Disorder cases (n=7,603) n (%)	Controls (n=75,782) n (%)
Parcel centroid	3578 (47.1)	34658 (45.7)
Uniform lot interpolation	1537 (20.2)	14865 (19.6)
Address range interpolation	2054 (27.0)	21375 (28.2)
Zip code tabulation area centroid, city centroid, or county subdivision centroid	425 (5.6)	4737 (6.2)
Not geocoded	9 (0.1)	147 (0.2)

Supplemental Material, Table S2. Exclusion criteria used when estimating pregnancy period specific monitor-based air pollution exposure metrics.		
Pollutant	Data Availability	Criteria
CO	hourly	*Criteria for hourly measurements:*<br>At least 50% of hourly values available per 24-hr period and at least 50% of hourly values available from 6am-6pm. If sufficient data were available, a daily (24-hour) average was generated based on the hourly data.
		Criteria for pregnancy periods:<br>We required at least 15 readings for each full month in a given period (trimester or entire pregnancy) as well as 15 readings during the last 30 days of the pregnancy period.
NO, NO_2,	hourly	*Criteria for hourly measurements:*<br>At least 50% of hourly values available per 24-hr period and at least 50% of hourly values available from 8am-8pm.
		Criteria for pregnancy periods:<br>We required at least 15 readings for each full month in a given period (trimester or entire pregnancy) as well as 15 readings during the last 30 days of the pregnancy period.
O_3 (10am-6pm)	hourly	*Criteria for hourly measurements:*<br>At least 50% of hourly values available from 10am-6pm.
		Criteria for pregnancy periods:<br>We required at least 15 readings for each full month in a given period (trimester or entire pregnancy) as well as 15 readings during the last 30 days of the pregnancy period.
PM_{10}	24-hour average, every 6 days	*We required 3 or more values to be available per each full pregnancy month and during the last 30 days of pregnancy.*
$PM_{2.5}$	24-hour average, every 3 days	*We required 5 or more values to be available per each full pregnancy month and during the last 30 days of pregnancy.*

 http://dx.doi.org/10.1289/ehp.1205827

Supplemental Material, Table S3. Pollutant distributions and Pearson's correlation coefficients for entire pregnancy averages in controls

					Pearson's Correlation Coefficients									
					U-LUR[b]		S-LUR[c]		Criteria Pollutants					
Pollutant[a]	n	Mean	IQR	SD	LUR - NO	LUR- NO_2	LUR - NO	LUR- NO_2	CO	NO	NO_2	Ozone	PM_{10}	$PM_{2.5}$
U-LUR - NO[b]	75623	22.4	9.40	8.3	1.00									
U-LUR- NO_2[b]	75623	22.9	5.41	4.5	0.81	1.00								
S-LUR - NO[c]	64129	28.7	18.46	14.7	0.77	0.62	1.00							
S-LUR- NO_2[c]	64128	28.0	9.70	7.3	0.66	0.70	0.71	1.00						
CO	75565	1.0	0.55	0.5	0.27	0.26	0.56	0.53	1.00					
NO	75565	39.2	29.67	20.5	0.37	0.31	0.70	0.44	0.78	1.00				
NO_2	75565	30.8	10.47	7.6	0.35	0.43	0.58	0.73	0.77	0.73	1.00			
Ozone	75565	36.8	11.54	8.9	-0.33	-0.23	-0.57	-0.34	-0.55	-0.73	-0.50	1.00		
PM_{10}	69263	36.3	8.25	6.1	0.11	0.23	0.30	0.55	0.42	0.28	0.57	-0.17	1.00	
$PM_{2.5}$	59483	19.6	4.68	3.5	0.22	0.26	0.45	0.52	0.60	0.58	0.65	-0.47	0.58	1.00

[a] Pollutant values are expressed in the following units: CO ppm; NO, NO_2, ppb; PM, $\mu g/m^3$

[b] Unseasonalized Land Use Regression

[c] Seasonalized Land Use Regression

Supplemental Material, Table S4. Associations between trimester average air pollution exposure during pregnancy and Autistic Disorder, conditional logistic regression analysis using matched controls

		Adjusted[a]	
Exposure Metric	IQR[b]	N (case/control)	OR (95% CI)
S-LUR-NO, 1st trimester	18.5ppb	6281/52173	1.01 (0.98, 1.04)
S-LUR-NO, 2nd trimester	18.5ppb	6281/52173	1.01 (0.98, 1.04)
S-LUR-NO, 3rd trimester	18.5ppb	6246/51897	0.99 (0.96, 1.03)
S-LUR-NO_2, 1st trimester	9.7ppb	6281/52173	1.03 (0.98, 1.08)
S-LUR-NO_2, 2nd trimester	9.7ppb	6281/52173	1.03 (0.98, 1.08)
S-LUR-NO_2, 3rd trimester	9.7ppb	6246/51897	1.04 (0.98, 1.09)
CO, 1st trimester	0.55ppm	7421/72253	1.01 (0.98, 1.05)
CO, 2nd trimester	0.55ppm	7421/72253	0.99 (0.95, 1.02)
CO, 3rd trimester	0.55ppm	7383/71912	0.98 (0.94, 1.02)
NO, 1st trimester	29.67ppb	7421/72253	1.02 (0.99, 1.05)
NO, 2nd trimester	29.67ppb	7421/72253	1.00 (0.97, 1.03)
NO, 3rd trimester	29.67ppb	7383/71912	0.98 (0.95, 1.02)
NO_2, 1st trimester	10.47ppb	7421/72253	1.04 (0.99, 1.08)
NO_2, 2nd trimester	10.47ppb	7421/72253	1.01 (0.97, 1.06)
NO_2, 3rd trimester	10.47ppb	7383/71912	1.02 (0.97, 1.07)
Ozone (O_3), 1st tri	11.54ppb	7421/72253	1.00 (0.97, 1.03)
Ozone (O_3), 2nd tri	11.54ppb	7421/72253	1.02 (1.00 1.05)
Ozone (O_3), 3rd tri	11.54ppb	7383/71912	1.04 (1.01, 1.06)
PM_{10}, 1st trimester	8.25μg/m^3	6795/63662	1.00 (0.96, 1.05)
PM_{10}, 2nd trimester	8.25μg/m^3	6795/63662	1.01 (0.97, 1.06)
PM_{10}, 3rd trimester	8.25μg/m^3	6752/63259	1.02 (0.98, 1.06)
$PM_{2.5}$, 1st trimester	4.68μg/m^3	5840/55776	1.04 (0.99, 1.08)
$PM_{2.5}$, 2nd trimester	4.68μg/m^3	5840/55776	1.02 (0.98, 1.06)
$PM_{2.5}$, 3rd trimester	4.68μg/m^3	5811/55512	1.03 (0.99, 1.08)

[a] Adjusted for: maternal age, education, race/ethnicity, maternal place of birth; type of birth, parity, insurance type, gestational weeks at birth (continuous)
[b] Pollutant-specific entire-pregnancy IQR used as standard for trimester specific IQR
